# Generation of genome-scale gene-associated SNPs in catfish for the construction of a high-density SNP array

**DOI:** 10.1186/1471-2164-12-53

**Published:** 2011-01-21

**Authors:** Shikai Liu, Zunchun Zhou, Jianguo Lu, Fanyue Sun, Shaolin Wang, Hong Liu, Yanliang Jiang, Huseyin Kucuktas, Ludmilla Kaltenboeck, Eric Peatman, Zhanjiang Liu

**Affiliations:** 1 The Fish Molecular Genetics and Biotechnology Laboratory, Department of Fisheries and Allied Aquacultures and Program of Cell and Molecular Biosciences, Aquatic Genomics Unit, Auburn University, Auburn, AL 36849, USA; 2 The Shellfish Genetics and Breeding Laboratory, Fisheries College, Ocean University of China, Qingdao, 266003, PR China

## Abstract

**Background:**

Single nucleotide polymorphisms (SNPs) have become the marker of choice for genome-wide association studies. In order to provide the best genome coverage for the analysis of performance and production traits, a large number of relatively evenly distributed SNPs are needed. Gene-associated SNPs may fulfill these requirements of large numbers and genome wide distribution. In addition, gene-associated SNPs could themselves be causative SNPs for traits. The objective of this project was to identify large numbers of gene-associated SNPs using high-throughput next generation sequencing.

**Results:**

Transcriptome sequencing was conducted for channel catfish and blue catfish using Illumina next generation sequencing technology. Approximately 220 million reads (15.6 Gb) for channel catfish and 280 million reads (19.6 Gb) for blue catfish were obtained by sequencing gene transcripts derived from various tissues of multiple individuals from a diverse genetic background. A total of over 35 billion base pairs of expressed short read sequences were generated. Over two million putative SNPs were identified from channel catfish and almost 2.5 million putative SNPs were identified from blue catfish. Of these putative SNPs, a set of filtered SNPs were identified including 342,104 intra-specific SNPs for channel catfish, 366,269 intra-specific SNPs for blue catfish, and 420,727 inter-specific SNPs between channel catfish and blue catfish. These filtered SNPs are distributed within 16,562 unique genes in channel catfish and 17,423 unique genes in blue catfish.

**Conclusions:**

For aquaculture species, transcriptome analysis of pooled RNA samples from multiple individuals using Illumina sequencing technology is both technically efficient and cost-effective for generating expressed sequences. Such an approach is most effective when coupled to existing EST resources generated using traditional sequencing approaches because the reference ESTs facilitate effective assembly of the expressed short reads. When multiple individuals with different genetic backgrounds are used, RNA-Seq is very effective for the identification of SNPs. The SNPs identified in this report will provide a much needed resource for genetic studies in catfish and will contribute to the development of a high-density SNP array. Validation and testing of these SNPs using SNP arrays will form the material basis for genome association studies and whole genome-based selection in catfish.

## Background

Single nucleotide polymorphisms (SNPs) are alternative bases at any given position of DNA. They are among the most abundant type of genetic variations and widely distributed within genomes. Theoretically, SNPs can have four alleles in the population, but they most often exist as bi-allelic markers. Because of their potential for high genotyping efficiency, automation, data quality, genome-wide coverage and analytical simplicity [1], SNPs have rapidly become the marker of choice for many applications in genetics and genomics. In particular, SNPs are most suitable for whole genome association studies because linkage disequilibrium can be detected with high density SNP coverage of the genome when working with performance and production traits. For instance, simultaneous analysis of thousands of SNPs have enabled genome-wide association studies for complex traits in chicken [2], pig [3,4] cattle [5-7] horse [8] and sheep [9,10]. However, such studies have not been possible with most aquaculture species including catfish because large numbers of SNPs have not been available.

In species where the whole genome has been sequenced, SNPs have been identified from genome sequencing efforts. In most cases, SNPs were identified by sequence variations between the two alleles of a single diploid individual whose genome was sequenced [11]. More recently, the identification of SNPs in non-model species has been fuelled by mining large numbers of expressed sequence tags (ESTs) available in many species. Likewise, gene-associated SNPs derived from ESTs have been identified in several fish species, including Atlantic salmon [12], Atlantic cod [13] and catfish [14-16]. In spite of being relatively effective, SNP identification from ESTs is limited by sequence coverage and depth. For instance, of the 303,000 putative SNPs identified from catfish ESTs, only 48,594 were identified from contigs containing at least four ESTs and at least two sequences bearing the minor allele. A majority of the catfish EST contigs (56% of 45,306) contain only two or three sequences [16]. Putative SNPs identified from such contigs would have the minor alleles represented by only one sequence. Such SNPs could represent sequence errors and therefore, are not reliable [15].

To identify larger numbers of gene-associated SNPs, higher throughput expressed sequence reads are needed to increase coverage and depth and ensure sequence accuracy. Next generation sequencing technologies such as Roche/454, Illumina/Solexa, and ABI/SOLiD sequencing platforms are particularly adapted to producing high coverage of expressed sequences within contigs [17]. Transcriptome analysis using next generation sequencing with multiple individuals has been demonstrated to be very effective for SNP identification [18]. Recently, 454 sequencing was applied for the identification of gene-derived SNPs in a number of species such as eucalyptus grandis [18], pine tree [19], butterfly [20], lake sturgeon [21] and coral [22].

While the 454 sequencing technology has been widely used for transcriptome analysis, Illumina sequencing technology is being gradually accepted for its dramatically improved sequencing throughput and quality [23,24]. Paired-end sequencing technology along with the longer sequence reads make it possible to assemble contigs of transcripts from Illumina short reads. Such assemblies are aided by the presence of reference genome and/or reference transcriptome sequences [19,25]. In this context, a large number of ESTs of catfish are available. The objective of this study is to conduct transcriptome sequencing from multiple individuals of both channel catfish (*Ictalurus punctatus*) and blue catfish (*I. furcatus*) in order to identify gene-associated SNPs for the development of SNP arrays in catfish.

## Results

### Generation of expressed short reads

Illumina sequencing was conducted to generate short sequence reads of expressed sequences. Two cDNA libraries were made from pooled RNA samples prepared from a total of 11 tissues of 47 channel catfish and 19 blue catfish, respectively, representing major strains used in commercial production. The cDNAs were sequenced with one lane each using Illumina GA-II and Illumina HiSeq 2000 that generated 48.6 million 36-bp paired-end reads and 173.9 million 100-bp paired-end reads for channel catfish, and 66.9 million 36-bp paired-end reads and 216.6 million 100-bp paired-end reads for blue catfish (Table 1). After removal of ambiguous nucleotides, low-quality sequences (quality scores <20) and sequences less than 15 bp, sequences totaling 15.6 billion base pairs for channel catfish and 19.6 billion base pairs for blue catfish were generated (Table 1).

**Table 1:** Summary of Illumina expressed short reads production and filtration

Catfish species	No. of tissues	No. of fish	Sequencer	Sequence length*	**Reads (X10**^**6**^**)**	**Bases sequenced (X10**^**9**^**)**	**Reads after trimming (X10**^**6**^**)**	**Bases after trimming (X10**^**9**^**)**
Channel	11	47	Illumina GA-II	36 bp	48.6	1.8	47.2	1.7
			HiSeq 2000	100 bp	173.9	17.4	171.6	13.9

Blue	11	19	Illumina GA-II	36 bp	66.9	2.3	62.1	2.2
			HiSeq 2000	100 bp	216.6	21.7	212.5	17.4

Total	-	-	-	-	506.0	43.2	493.4	35.2

Eleven tissues were used for RNA preparation including brain, gill, head kidney, intestine, liver, muscle, skin, spleen, stomach, heart, and trunk kidney. *Paired-end reads were generated in different lengths of either 36 bp or 100 bp as a result of different sequencers, Illumina GA-II or HiSeq 2000.

### Assembly of the expressed short reads

Assembly of the expressed short reads was conducted in several ways. First, reference assemblies of channel catfish expressed short reads and blue catfish expressed short reads were conducted separately using all existing catfish ESTs as a reference. Such assemblies would allow establishment of contigs for channel catfish expressed short reads and blue catfish expressed short reads separately to allow identification of intra-specific SNPs that are anchored (scaffold) by longer EST reference sequences. Such an assembly is superior to the total *de novo *assembly of the expressed short reads which generates very large numbers of contigs, over 800,000 (data not shown). As shown in Table 2, over two thirds of the expressed short reads were assembled with the reference assemblies. Over 152 million reads of channel catfish (69.8%) and 183 million reads of blue catfish (66.7%) were assembled into 103,650 and 104,475 contigs, respectively. The contigs were reasonably long with an average contig length of 670 bp and 775 bp, respectively, for channel catfish and blue catfish (Table 2).

**Table 2:** Summary of reference assembly of expressed short reads of channel catfish and blue catfish

Catfish species	No. of reads used for assembly	No. of reads assembled	% sequences assembled	No. of contigs	Average contig length (bp)	Average contig size^*^	**Average coverage**^**#**^
Channel	218.8 × 10^6^	152.6 × 10^6^	69.8%	103,650	670	1,473	137.4
Blue	274.6 × 10^6^	183.8 × 10^6^	66.7%	104,475	775	1,760	164.2

*Number of reads per contig. ^#^Total number of assembled read bases/Total number of bases in consensus sequence.

Despite generating an efficient reference assembly, over 66 million channel catfish reads and 90 million blue catfish reads were not assembled with the reference assembly. These reads could represent additional genes that were not represented by the EST reference sequences, or they could come from gene regions that were not represented by the EST references. In order to make them useful resources for SNP identification, *de novo *assembly of these remaining reads was conducted. As shown in Table 3, over 70% of these unassembled expressed short reads could be assembled *de novo*, generating 420,165 contigs and 420,953 contigs for channel catfish and blue catfish, respectively. However, the average contig length was much shorter than those in the reference assembly, with 298 bp and 315 bp for channel catfish and blue catfish, respectively. These contigs are also useful resources for the identification of intra-specific SNPs.

**Table 3:** Summary of *de novo *assembly of the unassembled expressed short reads from reference assembly of channel catfish and blue catfish

Catfish species	No. of reads used for assembly	No. of reads assembled	% sequences assembled	No. of contigs	Average contig length (bp)	Average contig size*	**Average coverage**^**#**^
Channel	66.2 × 10^6^	46.8 × 10^6^	70.7%	420,165	298	111	19.7
Blue	90.8 × 10^6^	64.3 × 10^6^	70.8%	420,953	315	153	26.4

All the newly generated expressed short reads were first assembled using reference assembly (Table 2), and those that were not assembled, i.e., they did not align *in silico *to the existing catfish ESTs, were used for the *de novo *assembly. *Number of reads per contig. ^#^Total number of assembled read bases/Total number of bases in consensus sequence.

After separate analyses of channel catfish and blue catfish expressed short reads, reference and *de novo *assemblies were conducted using combined channel catfish and blue catfish expressed short reads in order to identify inter-specific SNPs. A total of 493.4 million expressed short reads from both channel catfish and blue catfish (all catfish) were used. The reference assembly of all catfish expressed short reads placed 336.0 million reads (68%) into 104,870 contigs, with an average contig length of 686 bp. Similarly, the *de novo *assembly of all catfish expressed short reads generated 421,229 contigs, with an average contig length of 340 bp (Table 4). These contigs should be useful for the identification of inter-specific SNPs.

**Table 4:** Summary of assembly of all catfish expressed short reads

Assembly	No. of reads used for assembly	No. of reads assembled	% sequences assembled	No. of contigs	Avg. contig length	Max length	No. of large contigs (>1 kb)	Avg. contig size^*^	**Avg. coverage**^#^
Reference^1^	493.4 × 10^6^	336.0 × 10^6^	68.1%	104,870	686	6,849	17,756	3,204	330.8
*De novo*^2^	157.4 × 10^6^	107.2 × 10^6^	68.2%	421,229	340	4,615	4,133	255	44.1

^1^All expressed short reads from both channel catfish and blue catfish were first assembled using existing ESTs as references. ^2^Those that were not assembled into contigs with the reference ESTs were then assembled *de novo*. *Number of reads per contig. ^#^Total number of assembled read bases/Total number of bases in consensus sequence.

### Putative gene identity and annotation

Before SNP identification, we conducted analysis of putative gene identities to help assess how many genes may be included in the assemblies. To determine the putative gene identities, unique consensus sequences from the all catfish reference assembly and *de novo *assembly were searched against the Uniprot database and NCBI zebrafish Refseq protein database using BLASTX with a cutoff E-value of 1E-10. Of 104,870 all catfish contigs from the reference assembly, 32,350 (30.9%) had BLAST hits to the Uniprot database, and matched 17,766 unique protein accessions. As expected, a lower percentage of the contigs from *de novo *assembly had BLAST hits to Uniprot proteins. Of the 421,229 contigs, 24,168 (5.7%) had BLAST hits to the Uniprot database, with matches to 12,331 unique proteins. Altogether, of the 526,099 contigs, 56,518 (10.7%) had significant BLAST hits to the Unitprot database, and matched 24,440 unique protein accessions. Larger numbers of contig hits but fewer matches to unique proteins were observed when compared to the zebrafish Refseq protein database (Table 5). Altogether, 66,285 (12.6%) had BLAST hits to known proteins in zebrafish Refseq protein database that matched 19,899 unique protein accessions. This seemingly low percentage of contigs with BLAST hits is partially due to a high proportion of short contigs in the assembly of expressed short reads, although the percentage of the unique proteins of zebrafish hit by the unique catfish sequences in this study is comparable to levels reported in our previous catfish EST project [16]. Longer contigs were more likely to have BLAST hits to the annotated protein databases, 80% of our contigs with BLAST hits were over 350 bp in length (see Additional file 1), similar to observations in previous studies [16,18,19]. Nonetheless, BLAST searches identified a total of 24,440 unique protein accessions including 6,674 genes that were identified for the first time here from the catfish transcriptome.

**Table 5:** Summary of BLASTX searches to annotated protein databases

Assembly	Contigs hit Uniprot	% contigs with hits	Unique protein hits	Contigs hit zebrafish Refseq	% contigs with hits	Unique zebrafish Refseq hits
Reference	32,350	30.9%	17,766	**36,597**	**34.9%**	**14,874**
*De novo*	24,168	5.7%	12,331	**29,688**	**7.1%**	**10,781**

Total	56,518	10.7%	24,440	**66,285**	**12.6%**	**19,899**

Contigs of two assemblies, the reference assembly with 104,870 contigs and the *de novo *assembly with 421,229 contigs, were used to search the Uniprot database and the zebrafish Refseq protein database to assess the number of related genes represented by catfish expressed sequences.

To assess the coverage of the catfish transcriptome achieved by our sequencing effort, the distribution of gene ontology (GO) annotations in catfish was compared with that of zebrafish.

**Figure 1:**
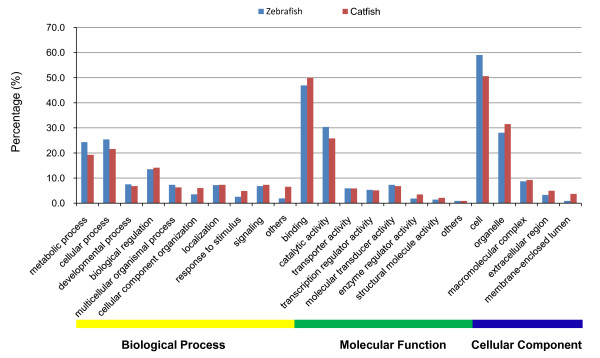
**Similarity of GO-term assignments for catfish and zebrafish genes**. Proportions of GO-terms assigned to annotated contigs from catfish assembly compared with the proportions found in the zebrafish genome annotation which serves as an indicator of the extent to which the catfish transcriptome has been characterized.

The unique genes from the catfish and the zebrafish annotated database were analyzed using generic GO-slim terms with Blast2GO [26,27]. The percentages of annotated catfish sequences assigned to GO-slim terms are very similar to those of zebrafish genes (Figure 1), suggesting a generally similar distribution of genes in different functional categories, and the depth of the coverage of the transcriptome.

### SNP identification

As summarized in Table 6, a total of 2,030,410 intra-specific putative SNPs were identified from the channel catfish sequence assembly; 2,497,806 intra-specific putative SNPs were identified from the blue catfish sequence assembly; and 4,236,135 putative SNPs were identified from the all catfish sequence assembly (intra-specific blue + intra-specific channel - intra-specific both + inter-specific). Almost two thirds of the putative SNPs were transitions.

**Table 6:** Summary of putative SNP identification from the catfish expressed short reads assembly

	Channel catfish	Blue catfish	All catfish
Contigs under analysis	523,815	525,428	526,099
Total SNPs	2,030,410	2,497,806	4,236,135
Transitions	1,311,220	1,616,477	2,751,244
Transversions	719,190	881,329	1,484,891
SNP/100 bp	1.6	1.8	3.0

Putative SNPs include all base variations involved in the sequence assemblies with at least four sequences present at the SNP position with minor allele sequences represented at least twice. *All catfish *represents both intra-specific and inter-specific SNPs. Note that the total SNPs from all catfish assembly is fewer than the sum of total SNPs from channel catfish and blue catfish due to shared SNP positions in the two catfish species.

Our previous research suggested that SNPs identified from contigs with at least four sequences at the SNP sites with the minor allele being represented at least twice are more reliable [15]. In this study, putative SNPs were further screened following specific criteria based on the read depth, minor allele frequency, the quality of flanking regions and absence of additional SNPs in the 15-bp flanking regions (see Methods). With these criteria, a total of 342,104 putative filtered SNPs were identified from channel catfish; 366,269 putative filtered SNPs were identified for blue catfish (Table 7); of these 25,143 putative filtered SNPs were identified from same positions in both channel catfish and blue catfish, while 420,727 putative filtered inter-specific SNPs were identified (Additional file 2). The number of intra-specific SNPs identified from same positions in both channel catfish and blue catfish may be underestimated, due to failure to capture sequences from one or both species in the current sequence data. A total of 146,573 filtered intra-specific SNPs in channel catfish were identified from positions where there were fewer than four blue catfish sequences, and similarly, 174,034 filtered intra-specific SNPs in blue catfish were identified from positions where there were fewer than four channel catfish sequences (see Additional file 2). Obviously, the failure to obtain sequences from one or both species at same positions would also cause the underrepresentation of inter-specific SNPs.

**Table 7:** Quality SNPs selected from the putative SNPs with a set of criteria as described in the Methods section

	Intra-specific SNPs		**Inter-specific SNPs**^**3**^
		
	**Channel catfish**^**1**^	**Blue catfish**^**2**^	
Total SNPs	342,104	366,269	420,727
Transitions	208,517	230,031	262,048
Transversions	133,587	136,238	158,679
No. of contigs with SNPs	168,458	190,197	232,972
No. of contigs with Uniprot hits & SNPs	28,067	30,376	32,515
No. of unique known genes containing SNPs	16,562	17,423	18,085

^1^SNPs identified at positions where there were SNPs within channel catfish; ^2^SNPs identified at positions where there were SNPs within blue catfish; ^3^SNPs identified at positions where there were no intra-specific channel catfish SNPs or intra-specific blue catfish SNPs, but the bases differed between the two species.

**Figure 2:**
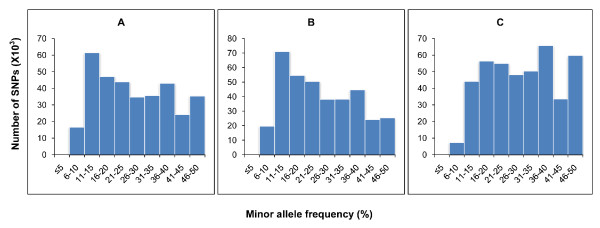
**Distribution of minor allele frequencies of SNPs identified for channel catfish, blue catfish and inter-species, as derived from analysis of sequence tags from the Illumina sequencing**. A: Intra-specific SNPs in channel catfish; B: Intra-specific SNPs in blue catfish and C: Inter-specific SNPs between the two species. The X-axis represents the SNP sequence derived minor allele frequency in percentage, while the Y-axis represents the number of SNPs with given minor allele frequency. Note that the majority of SNPs have minor allele frequencies more than 15%.

Since the information on minor allele frequency (MAF) is an important consideration in choosing which SNPs to be included in SNP arrays, the minor allele frequencies of SNPs in the discovery populations were estimated from the sequence data. As shown in Figure 2, the majority of SNPs have sequence derived minor allele frequencies more than 15%, and the average MAFs were 0.28, 0.26 and 0.31 in putative filtered SNPs identified for channel catfish, blue catfish and inter-species, respectively (Figure 2).

While the number of SNPs is important, their distribution in contigs and genes within the genome is also important when used for genetic analysis. A total of 168,458 channel catfish contigs and 190,197 blue catfish contigs were found to contain putative filtered SNPs, of which 13,414 contigs contain SNPs at same positions in both channel catfish and blue catfish. The number of unique Uniprot accessions hit by contigs containing SNPs was 16,562 for channel catfish, and 17,423 for blue catfish, suggesting that putative filtered SNPs were identified from the vast majority of catfish genes.

One important aspect of using the inter-specific hybrid system is to identify inter-specific SNPs. From this work, a total of 232,972 contigs were identified to contain 420,727 inter-specific SNPs, i.e., sequence variations between the two species, channel catfish and blue catfish. These SNPs were from at least 18,085 distinct genes as determined by unique hits to the Uniprot protein database (Table 7).

### Microsatellite markers identification

The 526,099 catfish contigs were surveyed to identify microsatellite markers. A total of 57,379 microsatellites were initially identified from 49,883 contigs. The majority of the microsatellites are dinucleotide repeats (Table 8). Of these microsatellites, 39,516 distributed within 34,539 contigs had sufficient flanking sequences on both sides for primer design. These microsatellites should be useful for genetic linkage mapping and other genetic studies.

**Table 8:** Summary of microsatellite markers identification from the all catfish expressed short reads assembly

Number of contigs of sequences surveyed	526,099
Number of contigs containing microsatellites	49,883
Total number of microsatellites identified	57,379
Di-nucleotide repeats	31,657
Tri-nucleotide repeats	16,925
Tetra-nucleotide repeats	8,235
Penta-nucleotide repeats	506
Hexa-nucleotide repeats	56
Number of microsatellites with sufficient flanking sequences	39,516
Number of contigs containing microsatellites with sufficient flanking sequences	34,539

### Assessment of SNP distribution

**Figure 3:**
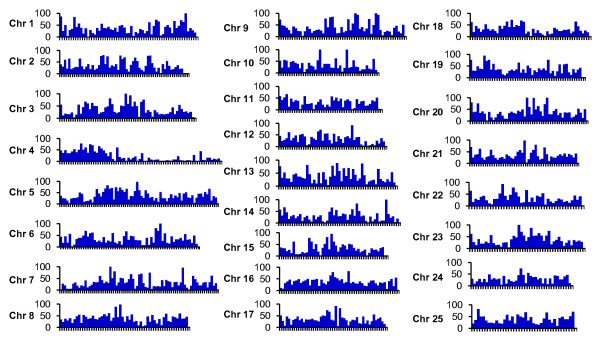
**Comparative analysis of the genes containing SNPs on 25 chromosomes of the zebrafish genome**. Each of the 25 zebrafish chromosomes was laid out in the X-axis with one million base pairs intervals, and the number of genes contained with filtered SNPs residing in the interval was plotted on the Y-axis.

SNPs distribution along the chromosomes of a genome is important for consideration of genome coverage using SNP markers. In the absence of a whole genome sequence assembly in catfish, we have taken a comparative genomic approach to plot the SNPs from expressed sequences onto the zebrafish genome sequence assembly. Contigs containing SNPs were used as queries against zebrafish transcripts to plot their putative genomic locations based on homology. As shown in Figure 3, the catfish expressed SNPs represent genes that are widely distributed along the chromosomes of all 25 zebrafish chromosomes. There are few gaps over one million base pairs in this comparative alignment (Figure 3).

## Discussion

In this work, we have conducted RNA-Seq analysis with pooled RNA samples from multiple individuals of both channel catfish and blue catfish to develop large numbers of high-quality SNPs. A total of 493.4 million reads allowed generation of a total of over 35 billion base pairs of expressed sequences. Previous to this report, a total of approximately 290 million base pairs of expressed sequences of catfish had been generated using traditional Sanger sequencing. This work represents more than 100 times more transcript sequences than the total previously submitted to GenBank. Our results demonstrate the efficiency and cost-effectiveness of next generation sequencing technologies in generating expressed sequences.

One great challenge of using Illumina sequencing for transcriptome analysis is the short read length. In this study, we have used both the Illumina GA-II and HiSeq 2000 sequencing platforms that generated read lengths of 36 bp or 100 bp. *De novo *assembly of the expressed short reads proved to be problematic even with gene-associated sequences. For instance, a total *de novo *assembly of the 218.8 million short reads from channel catfish would lead to over 800,000 contigs. Similarly, *de novo *assembly of 274.6 million short reads from blue catfish would lead to over 1,000,000 contigs. Such large numbers of short contigs may make subsequent applications of the EST or SNP resources less effective. However, such challenges are significantly alleviated when a large EST resource is available, as demonstrated by drastic reduction of contig numbers with the reference assembly in this study.

**Figure 4:**
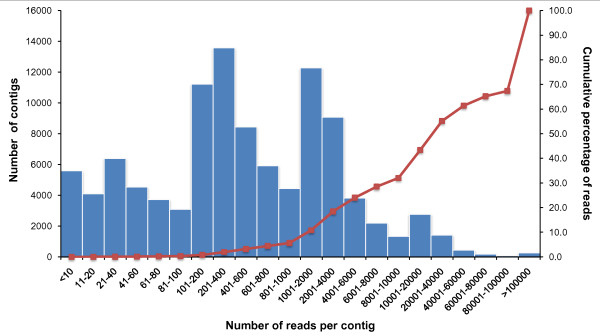
**Frequency of contigs of various sizes from the all catfish reference assembly**. The X-axis represents contig size (number of reads per contig). The curved line denotes the cumulative percentage of reads assembled. Note that a small number of very large contigs account for the majority of total reads. For instance, less than 0.3% of the contigs with over 100,000 reads per contig represent over 32% of all sequence reads assembled.

A second challenge is the over representation of highly expressed gene tags in transcriptome analysis. As shown in Figure 4, a small number of contigs (254) accounted for 32.6% of total reads. Clearly, there is a huge proportion of repeated sequencing and over representation of abundantly expressed genes. Obviously, this problem can be reduced by normalization of the cDNA. However, when a good EST reference is available, such a seemingly large problem is not as serious as the numbers indicate. As shown in Table 5, BLAST analysis of the reference assembly and *de novo *assembly contigs revealed that 24,440 unique Uniprot accessions were represented, suggesting that the expressed short reads provided good coverage of the catfish transcriptome. Additionally, previous, extensive EST sequencing of normalized and subtracted cDNA libraries resulted in 105,182 unique consensus sequences from channel catfish and blue catfish. Our sequencing here covered 104,870 of those contigs (99.7%), produced significant hits to 6,674 previously uncaptured genes, and covered thousands of additional transcript regions currently without annotation.

Pooling of RNA samples from multiple individuals followed by transcriptome analysis using next generation sequencing is among the most efficient methods for SNP identification. Through many years of efforts, a total of approximately 303,000 putative catfish SNPs were previously identified [16]. However, this study alone allowed identification of over 2 million SNPs from channel catfish and almost 2.5 million SNPs from blue catfish. This efficiency is even more obvious when considering filtered (high-quality) SNPs. While only 48,594 filtered SNPs were identified among all catfish ESTs [16], this work resulted in 342,104 filtered SNPs within channel catfish and 366,269 within blue catfish. In addition, more than 420,000 filtered SNPs were identified as inter-specific SNPs, and are valuable in genetics and breeding studies involving hybrid catfish.

One major challenge for SNPs is the problem caused by paralogous sequence variants (PSVs) and multisite sequence variants (MSVs) [28]. Putative SNPs detected may be false positives, potentially arising from sequencing errors or misassembly of PSVs or MSVs. Paralogs that share high levels of sequence similarity may have been assembled in the same contig due to the short read length of Illumina reads. A higher stringency of assembly may better discriminate between paralogs, but complete discrimination may prove to be difficult due to the lack of a reference genome sequence. On the other hand, a higher stringency of assembly would lead to the separate assembly of haplotypes from highly polymorphic genes [18]. Therefore, in order to select SNPs with high confidence, putative SNPs were screened based on several factors including surrounding sequence quality, absence of additional SNPs in the flanking regions, sequence depth and minor allele frequency. SNPs detected within contigs or regions of high sequence depth are more likely to be false positives. Therefore, setting a minimum minor allele frequency (e.g. 10%) for larger contigs may help reduce false SNP calling based on sequence errors. Additionally, multiple SNPs located close to one another (<15 bp) often represent sequence errors and prevent the design of primers and probes for SNP genotyping. A requirement of no additional SNPs in the 15-bp flanking region around a putative SNP was therefore applied.

Given the large numbers of SNPs generated that meet these minimal requirements, more stringent parameters can be applied in picking SNP sets for different applications. Average depth at putative SNP positions is greater than 100 sequences, providing high confidence in accuracy of identified SNPs within the pooled samples. Re-sequencing or limited validation of these samples by low-throughput SNP genotyping is costly and is unlikely to generate additional information. Ultimately, SNPs need to be validated by genotyping in a variety of reference mapping families and trait-selected populations using a high-density screening array. In catfish, the use of homozygous gynogenetic catfish [29] as controls will allow detection of false positives caused by PSVs or MSVs.

**Figure 5:**
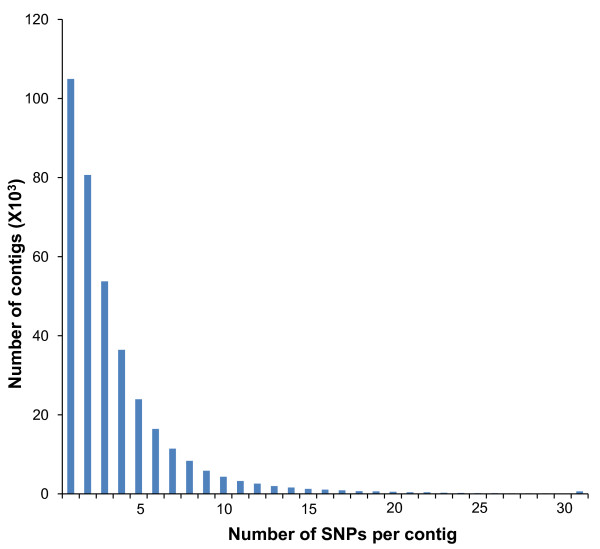
**Distribution of filtered SNPs per contig**. Histograms depict frequency of contigs with a given number of SNPs identified. Note that the majority of contigs have 5 or fewer SNPs per contig.

Genome-wide association studies of complex traits require a large number of SNPs. However, for research communities focused on non-model organisms, it is cost-prohibitive to genotype all SNPs in an association study with the throughput of current technologies. Selection of uniformly distributed SNPs across the genome for association studies is therefore very important [30]. Gene-associated SNPs identified in this study, as anticipated, appear to be widely distributed across the catfish genome based on comparative analysis with zebrafish. About 30% of all contigs with identified SNPs had one SNP and 66% had three or fewer SNPs per contig (Figure 5). In absence of a whole genome assembly, the assessment of the exact pattern of the SNP distribution in the catfish genome is not possible. However, when the contigs containing filtered SNPs were plotted to the zebrafish genome by BLAST analysis, they had a good coverage of all regions of all 25 zebrafish chromosomes (Figure 3). While chromosome breakage, fusions, and rearrangements between catfish and zebrafish have occurred during genome evolution, at the genomic scale it is reasonable to assume that these widely distributed genes in the zebrafish genome will have a similar genomic distribution in catfish.

## Conclusions

The approach to sample animals of diverse genetic backgrounds and sequence to sufficient depth for reliable SNP identification allowed the ability to detect many common SNPs across the entire genome. We have demonstrated that transcriptome analysis of pooled RNA samples from multiple individuals using Illumina sequencing technology is both technically efficient and cost-effective for generating expressed sequences. Such an approach is most effective when coupled to existing EST resources generated using traditional sequencing approaches because the reference ESTs facilitate effective assembly of the expressed short reads. The SNPs identified in this report will provide a much needed resource for genetic studies in the catfish scientific community and will contribute to the development of high density, cost-effective genotyping platforms. Validation and testing of SNPs using high-density arrays will subsequently lead to the production of a SNP array with well-spaced SNPs providing a powerful genotyping tool for the study of performance and production traits in catfish.

## Methods

### Sample and RNA isolation

Channel catfish of 47 individuals from five different aquaculture populations/fingerling sources (8 Marion Select, 10 Pearson, 11 Moyer, 10 Holland, 8 Noble) and blue catfish of 19 individuals from two different strains (7 Rio Grande and 12 D&B) were used for this study. Samples of 11 tissues including brain, gill, head kidney, intestine, liver, muscle, skin, spleen, stomach, heart, and trunk kidney were collected. The fish were euthanized with tricaine methanesulfonate (MS 222) at 300 mg/l before tissue collection. Tissue samples from each species were collected, pooled, immediately placed in 5 ml RNA later™ (Ambion, Austin, TX, USA) and kept at 4°C for 2-4 days until RNA extraction. Equal weight of each tissue from individuals of each species were combined, ground to a fine powder with mortar and pestle in the presence of liquid nitrogen and thoroughly mixed. A fraction of the tissue samples was used for RNA isolation. Total RNA was isolated using the RNeasy plus Mini Kit (Qiagen, Valencia, CA, USA) with DNase I (Invitrogen, USA) treatment following the manufacturer's protocol.

### Illumina sequencing

Sequencing was conducted commercially in HudsonAlpha Genomic Services Lab (Huntsville, AL, USA). Briefly, 100 ng of total RNA was used to prepare amplified cDNA using Ovation RNA-seq, a commercially available kit optimized for RNA sequencing (NuGEN Technologies, San Carlos, CA). The produced double-stranded cDNA was subsequently used as the input to the Illumina library preparation protocol starting with the standard end-repair step. The end-repaired DNA with a single 'A'-base overhang is ligated to the adaptors in a standard ligation reaction using T4 DNA ligase and 2 μM-4 μM final adaptor concentration, depending on the DNA yield following purification after the addition of the 'A'-base. Following ligation, the samples were purified and subjected to size selection via gel electrophoresis to isolate 350 bp fragments for ligation-mediated PCR (LM-PCR). Twelve cycles of LM-PCR were used to amplify the ligated material in preparation for cluster generation. For each species of channel catfish and blue catfish, the prepared cDNA library was sequenced with 36-bp paired-end reads on one flow cell lane of the Illumina Genome Analyzer II platform and 100-bp paired-end reads on one flow cell lane of the Hiseq 2000 platform, respectively. The image analysis, base calling and quality score calibration were processed using the Illumina Pipeline Software v1.4.1 according to the manufacturer's instructions. Reads were exported in the FASTQ format and has been deposited at the NCBI Sequence Read Archive (SRA) under accession number SRA025099.

### Assembly of expressed short reads

**Figure 6:**
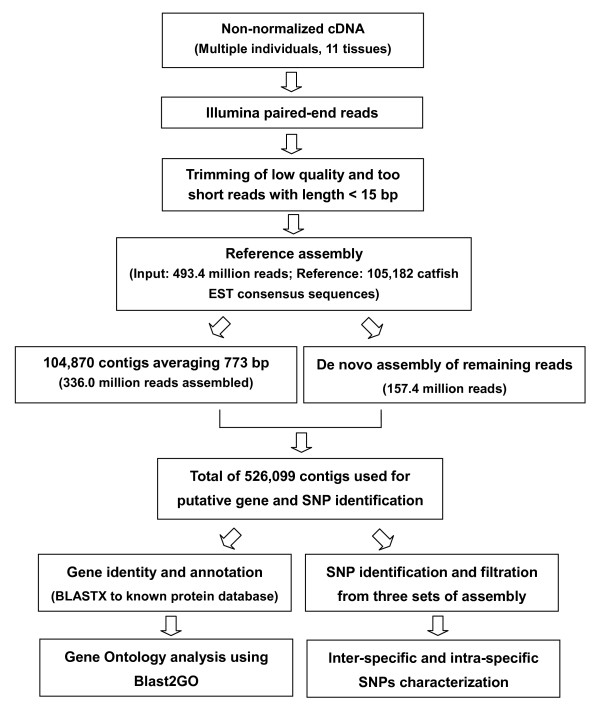
**Schematic presentation of the catfish transcriptome analysis**.

Sequence analysis was performed using the high-throughput sequencing module of CLC Genomics Workbench (version 4.0.2; CLC bio, Aarhus, Denmark). The raw reads were cleaned by trimming of adaptor sequences, ambiguous nucleotides ('N' in the end of reads) and low quality sequences with average quality scores less than 20. Trimmed reads less than 15 bp were also discarded from further analysis, the remaining reads were used in subsequent assembly. The approach of assembly in this study was based on a combination of reference assembly and *de novo *assembly. A reference-based assembly was firstly executed using a set of catfish unique sequences generated from ~500,000 Sanger-ESTs of both channel and blue catfish as a reference. For the reference assembly, the default local alignment settings were used to rank all potential matches, with mismatch cost of 2, deletion cost of 3 and insertion cost of 3. The highest scoring matches that shared ≥ 80% similarity with the reference sequence across ≥ 50% of their length were included in the alignment. This permissive alignment ensured that even reads derived from highly mutated orthologs between channel catfish and blue catfish would not be discarded. Reads that were not assembled into contigs in the reference assembly were entered into a subsequent *de novo *assembly with a higher stringency minimum match similarity (90%). Three separate assemblies were generated: channel catfish assembly, blue catfish assembly, and all catfish assembly (Figure 6).

### Gene identification and annotation

Unique consensus sequences from the all catfish assembly were compared against the Uniprot database and the zebrafish Refseq protein database (NCBI) using BLASTX (cutoff E-value of 1E-10) to obtain the putative gene identity. To estimate the proportion of annotated contigs that matched to unique genes in the known protein database, all BLASTX hits were filtered for redundancy in protein accessions. Assignment of Gene Ontology terms to annotated unique sequences was conducted using the program Blast2GO [26]. Ontology was categorized with respect to Biological Process, Molecular Function, and Cellular Component.

### SNP and microsatellite markers identification

Assembled contigs were scanned for SNPs utilizing SNP detection software included in CLC Genomics Workbench (CLC bio, Aarhus, Denmark). The central base quality score of ≥25 and average surrounding base quality score of ≥20 were set to assess the quality of reads at positions for SNP detection. Under the criteria of minimum coverage (read depth) of four and the minimum variant frequency of two, the variations compared to the reference sequence were counted as SNPs. Three lists of SNPs were generated from channel catfish, blue catfish and all catfish assembly, respectively. The identification of intra-specific SNPs for both channel and blue catfish, and inter-specific SNP between channel and blue catfish was achieved by comparing these three lists of SNPs. Inter-specific SNPs were defined as those that have sequence variations between channel catfish and blue catfish, but no sequence variations within channel catfish or within blue catfish; similarly, intra-specific SNPs were identified within channel catfish or within blue catfish; and intra-specific SNPs for both channel catfish and blue catfish were identified within both channel catfish and blue catfish at the same SNP position.

All the unique sequences were used to search for microsatellite makers using Msatfinder [31] with a repeat threshold of eight di-nucleotide repeats or five tri-, tetra-, penta-, or hexa- nucleotide repeats. The presence of at least 50-bp sequence on both sides of the microsatellite repeats were considered sufficient for primer design [32,33].

### Quality SNP screening

In order to identify quality SNPs, putative SNPs identified as mentioned above were further screened following specific criteria based on the read depth, minor allele frequency, the quality of flanking regions and absence of other SNPs within 15-bp flanking regions: only those SNPs with minor allele sequences representing no less than 10% of the reads aligned at the polymorphic loci were declared as quality SNPs; no extra SNPs or indels within 15-bp flanking regions were allowed; SNPs located in repetitive regions were also not considered. Potential repetitive elements were detected by RepeatMasker [34], SNPs located in repetitive regions were checked and ruled out using custom scripts. For practical application in SNP genotyping assays, only bi-allelic SNPs were considered in this study. To get a snapshot of the SNP distribution across the catfish genome, SNP- containing contigs with BLAST hits to the Ensembl zebrafish transcripts database were plotted along the zebrafish chromosomes.

## Authors' contributions

SL prepared the samples, conducted the bioinformatic analysis and was involved in writing the manuscript. ZZ, JL, FS, SW, HL, YJ, LK and HK were involved in one or more processes of samples collection, RNA extraction or bioinformatic analysis. EP provided assistance for fish collection, data analysis and manuscript preparation. ZL supervised the entire study and provided assistance for data analysis and manuscript preparation. All authors read and approved the final manuscript.

## Supplementary Material

Additional file 1 **Length distribution of contigs from the all catfish assembly with hits to the Uniprot database**. Click here for file

Additional file 2 **Categorization of different types of SNPs identified from the all catfish assembly**. (1) Intra-specific SNPs identified from positions where there were SNPs within channel catfish, but not within blue catfish; (2) Intra-specific SNPs identified from positions where there were SNPs within blue catfish, but not within channel catfish; (3) Intra-specific SNPs identified from positions where there were SNPs within both channel catfish and blue catfish; (4) Inter-specific SNPs identified from positions where there were no SNPs in channel catfish or in blue catfish, but the sequence differed between the two species; (5) Intra-specific SNPs identified from positions where there were SNPs within channel catfish and there were fewer than four blue catfish sequences; (6) Intra-specific SNPs identified from positions where there were SNPs within blue catfish and there were fewer than four channel catfish sequences. Intra-specific SNPs in channel catfish = (1) + (3) + (5); Intra-specific SNPs in blue catfish = (2) + (3) + (6); Intra-specific SNPs shared by the two species = (3); Inter-specific SNPs between the two species = (4). Click here for file
