# Supplementary figures and images for: Generation of genome-scale gene-associated SNPs in catfish for the construction of a high-density SNP array
Source: BMC Genomics. 2011 Jan 21;12:53. doi: 10.1186/1471-2164-12-53 (PMC3033819; doi:10.1186/1471-2164-12-53)

Number of contigs

Frequency

Cumulative %

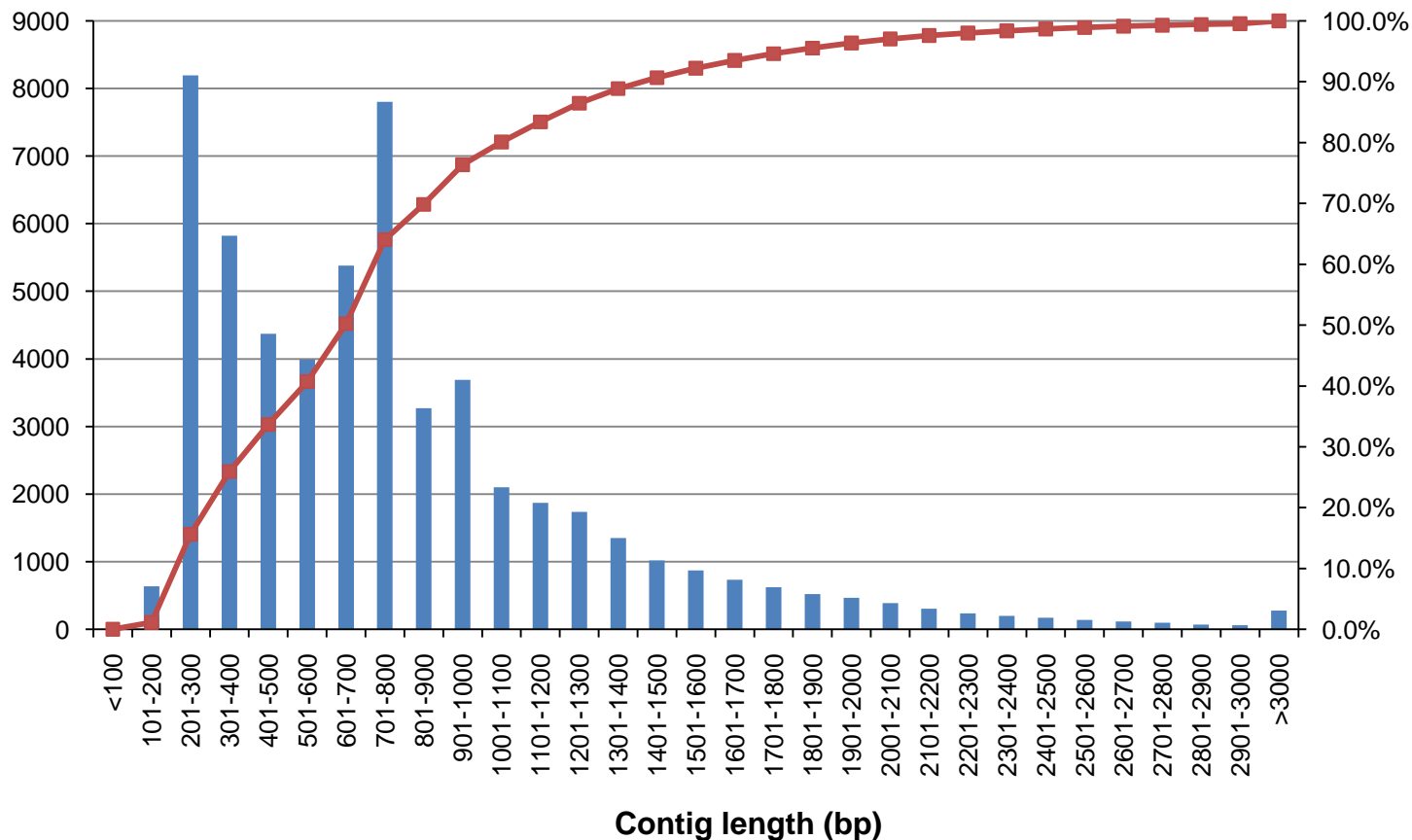

Supplement: Additional file 1 — Length distribution of contigs from the all catfish assembly with hits to the Uniprot database. [file 1471-2164-12-53-S1.PDF]
