# Supplementary material for: Generation of genome-scale gene-associated SNPs in catfish for the construction of a high-density SNP array
Source: BMC Genomics. 2011 Jan 21;12:53. doi: 10.1186/1471-2164-12-53 (PMC3033819; doi:10.1186/1471-2164-12-53)
Supplement: Additional file 2 — Categorization of different types of SNPs identified from the all catfish assembly. (1) Intra-specific SNPs identified from positions where there were SNPs within channel catfish, but not within blue catfish; (2) Intra-specific SNPs identified from positions where there were SNPs within blue catfish, but not within channel catfish; (3) Intra-specific SNPs identified from positions where there were SNPs within both channel catfish and blue catfish; (4) Inter-specific SNPs identified from positions where there were no SNPs in channel catfish or in blue catfish, but the sequence differed between the two species; (5) Intra-specific SNPs identified from positions where there were SNPs within channel catfish and there were fewer than four blue catfish sequences; (6) Intra-specific SNPs identified from positions where there were SNPs within blue catfish and there were fewer than four channel catfish sequences. Intra-specific SNPs in channel catfish = (1) + (3) + (5); Intra-specific SNPs in blue catfish = (2) + (3) + (6); Intra-specific SNPs shared by the two species = (3); Inter-specific SNPs between the two species = (4). [file 1471-2164-12-53-S2.PDF]

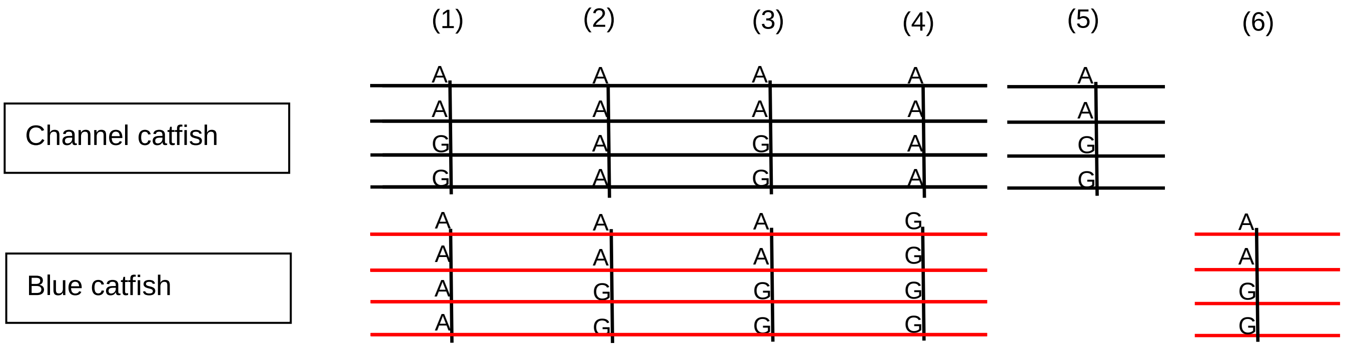

SNP categories	(1)	(2)	(3)	(4)	(5)	(6)
No. of SNPs	170,388	167,089	25,143	420,727	146,573	174,037
Transition	104,488	106,291	15,100	262,048	88,929	108,640
Transversion	65,900	60,798	10,043	158,679	57,644	65,397
No. of contigs *	100,716	97,057	13,414	232,972	93,462	117,488
No. of SNP-containing contigs with Uniprot hits **	21,997	22,432	2,587	32,515	13,865	14,902
No. of unique known genes with SNPs ***	14,048	14,304	2,178	18,085	10,162	10,460

Notes:

*: Number of contigs which each category of SNPs were indentified from.

**: Number of Uniprot hits of contigs which each category of SNPs were indentified from.

***: Number of unique genes which each category of SNPs were indentified from.
